# Experimental studies of the effects of hydrophobic coatings on flow separation around 3D bluff bodies

**DOI:** 10.1186/s42774-025-00211-w

**Published:** 2025-12-30

**Authors:** Naseeb Ahmed Siddiqui, Martin Agelin-Chaab

**Affiliations:** 1 Engineering Cluster, Singapore Institute of Technology, Singapore, 828608 Singapore; 2 Faculty of Engineering and Applied Science, Ontario Tech University, Oshawa, L1H 7K4 Canada

**Keywords:** Hydrophobic surfaces, Bluff body, Drag reduction, Flow control

## Abstract

This study explores how hydrophobic coating influences the structure of the flow separation at the rear end of 3D bluff bodies. Two 3D representative bluff bodies, namely the standard Ahmed body (SAB), which features flow separation and reattachment at its slant surface, and the elliptical Ahmed body (EAB), which exhibits fully separated flow, are employed. The bluff bodies were coated with Ultra Ever Dry hydrophobic paint. Experiments were carried out on pairs of coated and uncoated SABs and EABs in a water tunnel utilizing time-resolved and standard particle image velocimetry (PIV) at a Reynolds number of 4.3 × 10^4^ based on the model height. The results show that hydrophobic coatings influence the flow features of these bluff bodies. For the SAB, the coating alters the slant separation bubble, increasing the reattachment length by 80% and reducing the shear stress. The Strouhal number on the slant surface of the SAB also increases, with a dominant value of *S*_*t*_ = 0.24. Proper orthogonal decomposition (POD) analysis shows dominant Strouhal numbers of *S*_*t*_ = 0.36 and *S*_*t*_ = 0.48 for the first and second POD modes, respectively. Additionally, dynamic mode decomposition (DMD) analysis identifies a dominant Strouhal number of *S*_*t*_ = 0.3 in the wake. Conversely, the EAB, which already has a fully separated flow, is less affected by the coating. The wake recirculation length is reduced by 6%. Strouhal numbers on the coated EAB’s slant surface range from 0.40 to 0.55, and those in the wake vary from 0.25 to 0.85. The POD analysis does not reveal dominant Strouhal numbers in the EAB’s wake, while the DMD analysis indicates a dominant Strouhal number of *S*_*t*_ = 0.013, pointing to energetic modes due to the fully separated flow. These findings demonstrate that hydrophobic coatings affect the flow characteristics of 3D bluff bodies differently, depending on their inherent flow separation properties.

## Introduction

Complex flow phenomena, such as turbulent flows over complex three-dimensional (3D) bluff bodies, are important in both academic research and industrial applications [1]. Several solutions have been proposed to control the turbulent flow structure around bluff bodies. These include active methods (such as blow/suction and gas injection) and passive devices (flap, splitter plate, and vortex generators) [2]. The primary mechanism is either changing the wake structure or delaying the flow separation. The downside of these techniques is that active techniques require input energy, whereas passive devices are ineffective at off-design conditions.

Recently, flow control using hydrophobic coatings has achieved significant attention. These coatings modify the surface characteristics to influence the flow. A large Eddy simulation (LES) study on a 2D backward-facing step with the hydrophobic effect was analyzed. An increase of 4% in the reattachment length and 3% in the recirculation length were observed [3]. Additionally, in the experimental study by Muralidhar et al. [4], ridges were used at Reynolds numbers up to 10,000. The flow was sensitive to the ridge alignment, which is fabricated using lithographic techniques. Ridges parallel to the flow increased the shedding frequency compared to those aligned normally. The onset of shedding was reduced while the recirculation region increased. Similarly, a circular cylinder oscillation induced by hydrophobic coating was measured between Reynolds numbers 1300 and 2300. The coatings reduced the amplitude and lift of the oscillating cylinder. Furthermore, the recirculation bubble grew in both length and width, but vortex intensity and lift coefficient decreased [5].

Brennan et al. [6] studied drag reduction in a circular cylinder at Reynolds numbers up to $1.4\times {10}^{4}$. They reported a 28% drag reduction in the Cassie-Baxter state, where water does not penetrate surface peaks, compared to the Wenzel state, where it does [7]. In the study by Kim et al. [8], hydrophobic microparticles were sprayed on a cylindrical surface, while another cylinder was roughened with Teflon. The cylinder wake was studied based on the gas fraction, particle size, and direction of surface slip at Reynolds numbers of $0.7-23\times {10}^{3}$. Their results showed delayed separation and earlier vortex roll-up due to wake turbulence and shear layer effects. In a more recent study, Sooraj et al. [9] reported a drag reduction of 40% over hydrophobic hydrofoils at a 15° angle of attack. In another study, Sooraj et al. [10] analyzed the effect of hydrophobic coating over a circular cylinder. They observed increased turbulent kinetic energy (TKE) and Reynolds shear stresses. The onset of vortex shedding is also delayed, and a 15% drag reduction at an 860 Reynolds number is found. It was also highlighted that the hydrophobic coating affects the cylinder differently in various flow regimes.

The effect of the hydrophobic coating on 3D bluff bodies has found limited attention, except for simple bluff bodies such as spheres. For example, using LES, a sphere was studied by Zeinali and Ghazanfarian [3] at different Reynolds numbers based on the hydrophobic effect. They found a reduction in the drag coefficient by 25% and 46% at Reynolds numbers of 400 and 2000, respectively. Additionally, the Strouhal number is augmented by 25% due to the increased recirculation region. Another study by Jetly et al. [11] implemented the idea of hydrophobic coating on a metallic sphere in a water tank. With a small air layer on the surface due to the hydrophobic coating, approximately 80% drag reduction is achieved between Reynolds number 10^5^ and $3\times {10}^{5}$. This reduction was associated with the shifting of the separation point to the rear end, leading to decreased pressure drag. Recently, a DNS study conducted by Mollicone et al. [12] investigated the effect of hydrophobic coating around a 3D bump. This led to an almost 35% reduction in the recirculation region. The form drag was reduced due to a delay in the flow separation. The cause was ascribed to the significant changes in the turbulent kinetic energy production mechanism due to the hydrophobic coating.

In 3D bluff body investigations, the extensive literature available on the standard Ahmed body (SAB) is relevant [13]. The SAB shows complex three-dimensional turbulent structures dominated by the slant separation bubble (SSB), longitudinal C-vortices, and wake recirculation region. The SAB is characterized as having sharp flow transitions to different flow structures depending on the rear slant angle. The flow around some submarines produces similar flow features as the SAB at the rear end, which encounters significant resistance in water [14, 15]. There are other underwater applications of 3D bluff bodies, including underwater energy harvesting [16], big structures of bluff bodies, including caisson structures or oscillating water columns (OWC), wave energy converters (WECs) [17] and underwater energy storage [18]. Therefore, studying hydrophobic coatings at the rear end flow features of the SAB has tremendous significance in the bluff body flow control domain, especially in underwater applications. Furthermore, a detailed study of the effect of hydrophobic coating around a 3D bluff body such as the SAB has not been studied, as far as the authors are aware. The studies are on simple bluff bodies such as cylinders [10, 19] and spheres [3, 11].

The objective of this study is to investigate the impact of hydrophobic coating on the complex flow structure of two representative 3D bluff bodies, specifically focusing on the nature of the flow separation. The study examines the SAB, which exhibits flow separation and reattachment, and a modified elliptical Ahmed body (EAB), characterized by fully separated flow at the rear end [20]. By analyzing the effects of hydrophobic coating in relation to flow separation, the study aims to enhance our understanding of its influence on turbulent flow over 3D bluff bodies, a topic with limited attention, particularly at the Reynolds number employed in this study. By applying hydrophobic coatings to the Ahmed body, this study leverages a well-understood model to explore new avenues of flow manipulation, making the findings both foundational and applicable to real-world scenarios. The choice of the Ahmed body thus not only facilitates accurate PIV measurements but aligns with the broader research goals of understanding and improving flow control through surface treatments. Hence, this research extends the use of the 3D Ahmed body to a new domain other than its traditional use.

The primary goal of this research is to improve understanding of how hydrophobic coatings influence turbulent flow dynamics and separation over 3D bluff bodies. By studying their effects on SAB and EAB, the research aims to develop effective flow control strategies that can optimize performance in engineering applications. This work seeks to address critical challenges in managing flow separation and turbulence to reduce drag and energy losses, which aligns with sustainability and energy efficiency goals. Additionally, the findings will highlight the potential of hydrophobic coatings for underwater systems and other practical applications. By bridging gaps in understanding surface-flow interactions and exploring innovative coating technologies, this research contributes to advancing the field of flow optimization and inspires more efficient system designs.

The experiments are conducted at Reynold number of 4.31 × 10^4^, based on the model height. It utilized both standard and time-resolved particle image velocimetry (PIV). The investigation characterizes the fields of the SAB and EAB, with and without the hydrophobic coatings. A detailed analysis of the mean velocity field, Reynolds stresses, frequency, proper orthogonal decomposition (POD), and dynamic mode decomposition (DMD) is conducted.

The remainder of the manuscript is organized as follows: Section 2 discusses the model geometry and experimental setup. Section 3 provides the results and discusses the effects of the hydrophobic coatings. Finally, Section 4 concludes the study with further recommendations for future work.

## Experimental setup

### Water tunnel

**Fig. 1:**
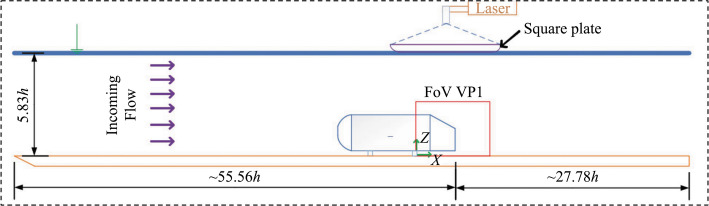
Schematic of the experimental setup. FOV is the field of view, and VP1 is vertical plane #1

An open water tunnel is used for the experiment. The test section has a depth of 0.45 m with a length and width of 6.00 m and 0.60 m, respectively. The walls of the test section are constructed from clear acrylic material for optical access. Before entering the test section, the flow goes through a series of flow conditioning devices, which feature a contraction ratio of 4.88:1. There are several types of mesh screens, hexagonal honeycombs, and perforated plates. The detailed experimental setup is reported in [20, 21] and will not be repeated here. Figure 1 shows a schematic representation of the experimental arrangement. Within the test section, the models are fixed on flat acrylic plates. The origin of the model axis is 55.56*h* from the inlet of the test section and 27.78*h* from the back end of the model (*h* is the model height).

Figure 1 displays the incoming flow direction and the laser system position at the top. The test model is placed within $\pm 1$ mm of the center of the test section during the test. At the entrance of the channel, 36-grit sandpaper is used to strip the incoming flow and speed up the transition to turbulent flow. The sandpaper is 100 mm long and covers the entire channel width (wall to wall). The measurement for the upstream boundary layer was made 29*h* from the tunnel inlet when the bluff body was removed.

### Model geometry and test conditions

**Fig. 2:**
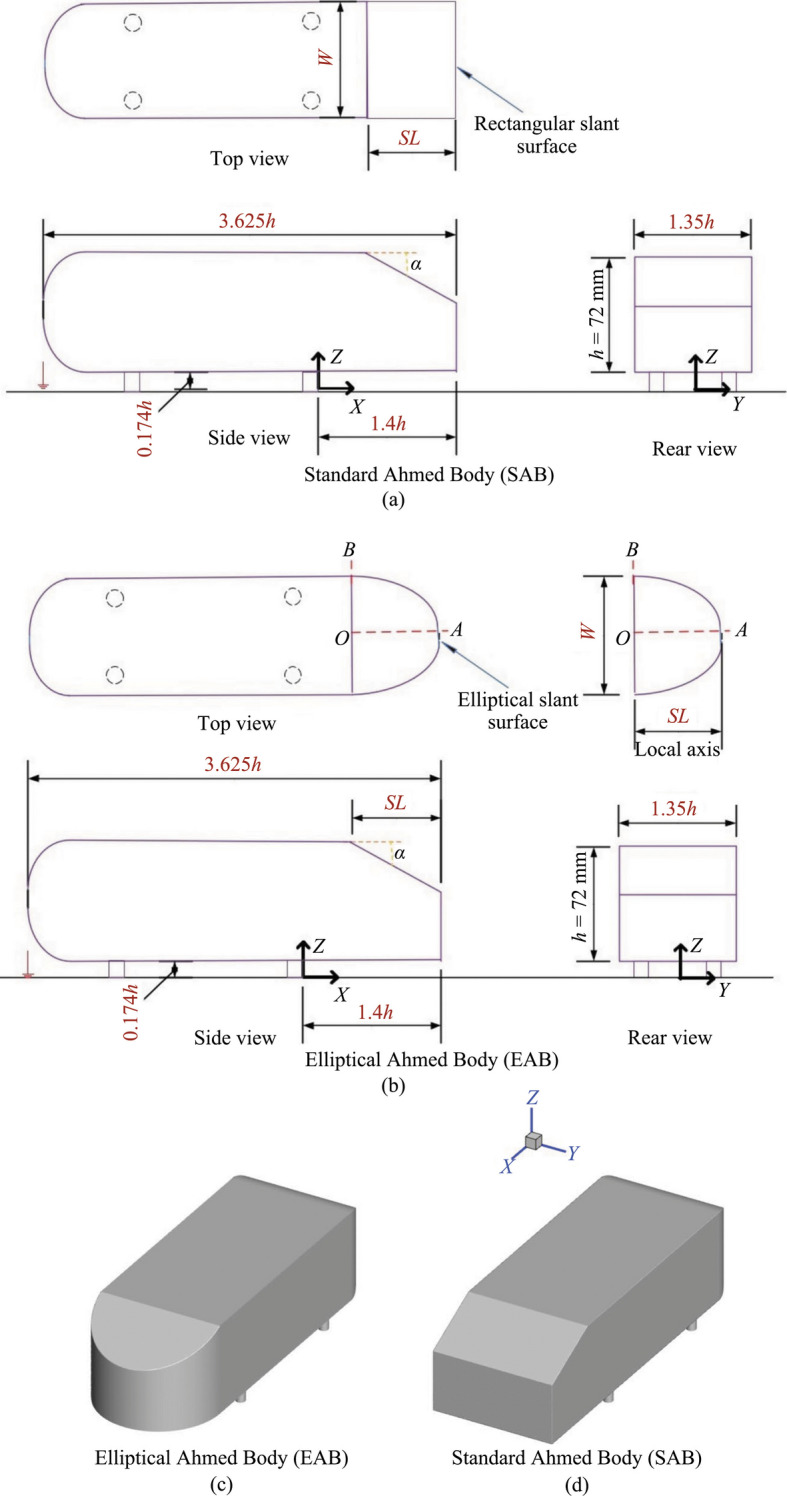
**a **Dimensions of the standard Ahmed body (SAB) and **b** elliptical Ahmed body (EAB) normalized by the model height, here *h* is the model height, *SL* is the slant length, *α* is the slant angle, and *W* is the width. The Local axis in **b** is shown to calculate the equation of the ellipse. The isometric views of the EAB and SAB are shown in **c** and **d**, respectively

A quarter scaled-down version of the 25° SAB is used in this investigation with a height *h* = 72 mm [13]. The general dimensions of the models are displayed in Fig. 2.

The water tunnel has a blockage ratio of 2.5%, which is significantly lower than the recommended 5% threshold value [22]. In Fig. 2, the elliptical slant surface represents the rear end of the EAB, and the rectangular slant surface identifies the SAB. More details about the bodies can be found in [20].

The models were 3D printed on the Makerbot *Z*-18 using white polylactic acid (PLA) in a fused deposition modeling process. The PLA filament had a diameter of 1.75 mm, a layer height of 0.2 mm, 35% infill, two top layers, and four bottom layers. The raster angle was 0° for the perimeter and 45° for the infill. Two SABs and two Elliptical Ahmed Bodies EABs were produced. To minimize surface glare, all models were painted with non-reflective black paint. Additionally, one SAB and one EAB were fully coated with Ultra Ever Dry, a commercial hydrophobic paint from UltraTech International Inc. (Florida, USA), following the supplier's instructions for applying multiple layers with bottom and top covers. The Keyence digital microscope, using the tangent method, measured the contact angle of water droplets on the surfaces. The contact angle on untreated PLA was approximately 91°, while on the hydrophobic coated surfaces, it was around 133°, confirming the coating's effectiveness.

The tests were carried out at a Reynolds number of $4.31\times {10}^{4}$ based on the model height (*h* = 72 mm) and the freestream velocity of *U*_∞_ = 0.60 m/s. The study is carried out at a room temperature of 25 °C. The related Froude number ${U}_{\infty }/\sqrt{gH}$ is 0.50, where *H* denotes the water depth.

### PIV measurement procedure

A high-speed diode-pumped dual-cavity dual-head laser is used to illuminate the flow field (Photonics DM30-527DH). The thickness of the laser sheet is oriented to about 1 mm using cylindrical and spherical lenses. The Phantom VEO-430L CMOS high-speed 12-bit cameras were used to capture the flow field. These cameras feature a resolution of 2560 × 1600 pixels and a pixel pitch of 10 µm. The observation is taken with a field of view (FOV) of 224 mm × 140 mm in the *X*–*Z* plane at a distance of 2 m from the inlet for the boundary layer characterization. Furthermore, the velocity field in the model symmetry plane (*X*–*Z* plane, *Y* = 0) was measured using two cameras with fields of view of 224 mm × 140 mm. The laser was used to illuminate the flow, and measurement in the horizontal plane (*X*–*Y*) was performed using a single camera.

A commercial software, Davis version 10 by LaVision Inc., was used to control the data collection. To perform time-resolved PIV measurements, a total of 48,000 images were acquired at a rate of 807 Hz. Additionally, measurements were made using the double-frame PIV (DF-PIV) to acquire 9000 pairs of images at a rate of 6 Hz. Because samples in DF-PIV are more statistically independent, they offer more precise assessments of the mean velocity and Reynolds stresses. The time-resolved (TR) PIV or TR-PIV was used to compute the time-dependent and spectral statistics. The DF-PIV was used to compute time-averaged statistics. The 4-pass correlation approach was used to calculate the velocity vectors. Initial interrogation areas (IAs) for the TR-PIV and DF-PIV were 128 × 128 pixels and 64 × 64 pixels, respectively, with a 50% overlap. Following earlier experiments, the final interrogation area was 24 × 24 pixels with 75% overlap [21]. The error analysis process in the current study was carried out in accordance with the guidelines by Casarsa and Giannattasio [23]. Consequently, the mean velocity uncertainty was calculated to be less than 2% at a 95% confidence level, and the Reynolds stress uncertainty was estimated to be 4% of their peaks. To calculate the measurement uncertainties in the mean streamwise velocity (*ξ*_*u*_), streamwise Reynolds normal stress (*ξ*_*uu*_), and Reynolds shear stress (*ξ*_*uv*_) for both the double frame and high-speed data, the following expressions are used:

1 $${\xi }_{u}={Z}_{c}{}^{{T}_{u}}\!\left/ \!{}_{\sqrt{N}}\right.,$$

2 $${\xi }_{uu}= {Z}_{c}\sqrt{\frac{1}{N}} \times (\frac{\overline{{u }^{\prime}{u}^{\prime}{u}^{\prime}{u}^{\prime}}}{{\left(\overline{{u }^{\prime}{u}^{\prime}}\right)}^{2}}-1),$$

3 $${\xi }_{uv}= {Z}_{c}\sqrt{\frac{1+{{\rho }^{2}}_{uv}}{N-1}},$$

4 $${\rho }_{uv}= \frac{\overline{u^{\prime}v^{\prime}}}{{u^{\prime}}_{rms}\times {v^{\prime}}_{rms}},$$

5 $${N}_{eff}=N\Delta t/2{T}_{int}.$$

From the expressions, *Z*_*c*_ is the confidence coefficient (value of 1.96 for 95% confidence level), ρuv represents the correlation coefficient, *N* is the total number of samples, and *N*_*eff*_ is the effective sample size. The time interval between successive images and the integral time scale is denoted by ∆*t* and *T*_*int*_. Similar expressions can be written for the uncertainties in the mean vertical velocity (*ξ*_*V*_) and vertical Reynolds normal stress (*ξ*_*vv*_).

## Results and discussions

This section provides a detailed description of the effects of the hydrophobic coatings. The time-averaged and time-dependent flow characteristics, such as the mean velocities, Reynolds stresses, and frequency spectrum, as well as POD and DMD techniques, were obtained and reported here to provide some insight. The analysis was carried out in the symmetry plane (*X*–*Z*) only. Based on the model height, the streamwise, spanwise, and wall-normal coordinates are normalized as follows:

6 $$X=\frac{x}{h}, Y=\frac{y}{h}, Z=\frac{z}{h}.$$

The components of velocity *x*, *y*, and *z* directions are:

7 $$U=\frac{u}{{U}_{\infty }}, V=\frac{v}{{U}_{\infty }}, W=\frac{w}{{U}_{\infty }},$$

where ${U}_{\infty }$ is the freestream velocity. The Reynolds stresses and Strouhal number is:

8 $${U}^{\prime}{U}^{\prime}=\frac{\overline{{u }^{\prime}{u}^{\prime}}}{{{U}_{\infty }}^{2}}, {U}^{\prime}{W}^{\prime}=\frac{\overline{{u }^{\prime}{w}^{\prime}}}{{{U}_{\infty }}^{2}},{W}^{\prime}{W}^{\prime}=\frac{\overline{w^{\prime}{w}^{\prime}}}{{{U}_{\infty }}^{2}},$$

9 $${S}_{t}=fh/{U}_{\infty },$$

where frequency is *f* and *h* represent the height of the model.

### Upstream flow characteristics

**Fig. 3:**
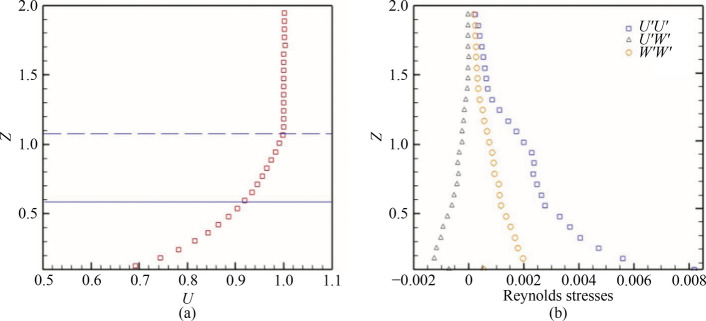
Profiles of the upstream flow **a** normalized streamwise velocity and **b** Reynolds stresses. The long-dotted line in **a** represents the thickness of the boundary layer, while the solid line indicates the mid-height of the test model. These quantities are extracted at *x* = 29*h* from the test section inlet

The profiles of the mean velocity and Reynolds stresses at the upstream flow are illustrated in Fig. 3. Figure 3a shows the extent of the model mid-height as a solid line with a long-dotted line denoting the boundary layer (*BL*). The model mid-height is observed to be within the *BL*. The displacement and momentum thickness were estimated as 0.15*h* and 0.12*h*. It is evident that the test model of the present study is within the boundary layer, which is similar to other water tunnel studies, such as [21, 24]. However, the near-field flow structure is not significantly affected because of the fixed separation points of the Ahmed body, except for the potential effect on the magnitude of the upper and low recirculation bubbles in the wake. Similarly, Fig. 3b reveals the averaged Reynolds stress profiles *U'U'*, *U'W'* and *W'W'*. It can be seen that the stresses converged to lower levels of *U'U'* = 0.004, *U'W'* = –0.0012, and *W'W'* = 0.0017 outside the boundary layer at about *Z* = 2.0.

### Mean velocity field

**Fig. 4:**
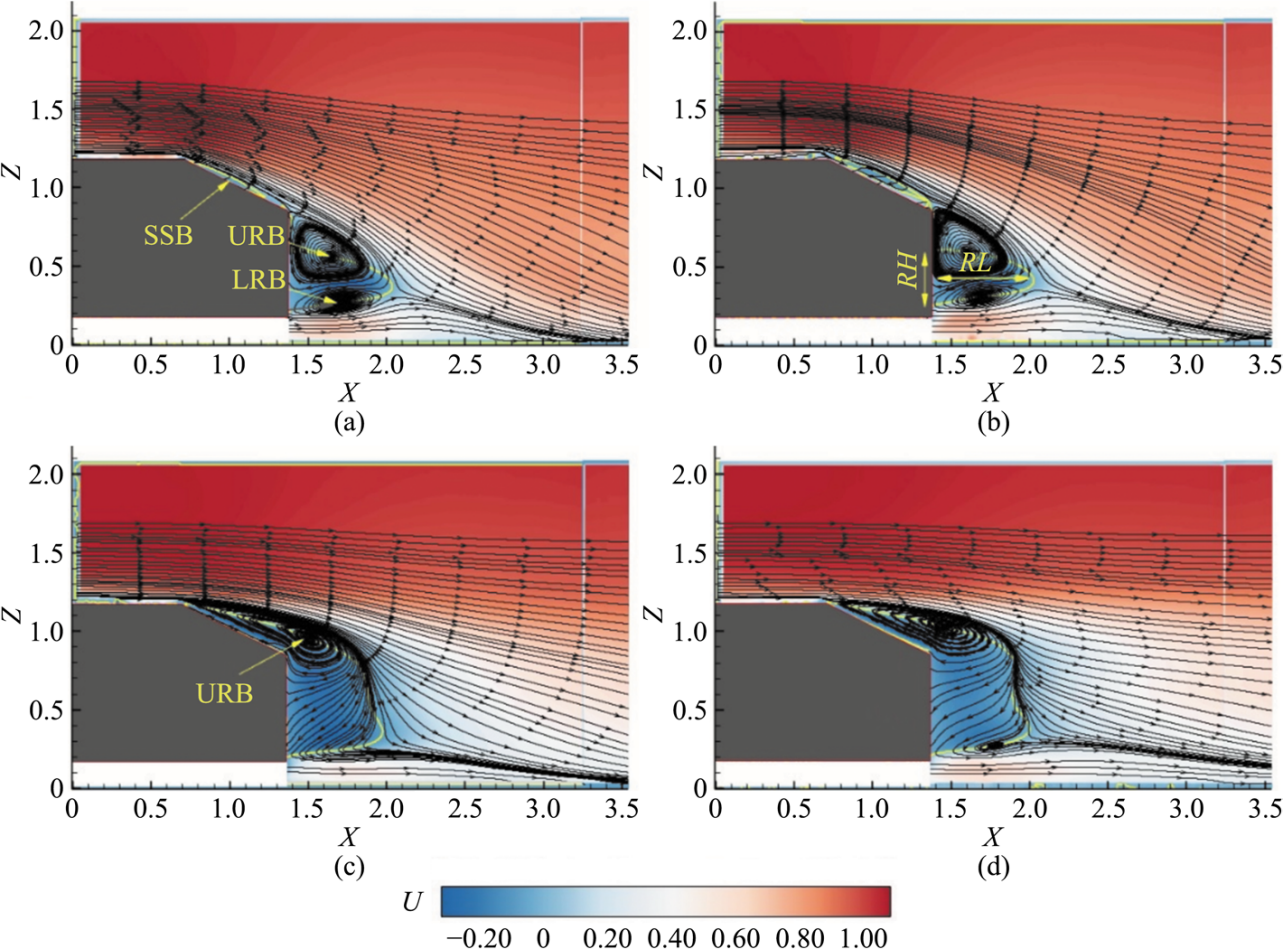
Time-averaged contours of the streamwise velocity (*U*) at the symmetry plane (*Y* = 0) superimposed on the mean streamlines: **a** SAB without the hydrophobic coating, **b** SAB with the hydrophobic coating, **c** EAB without the hydrophobic coating, and **d** EAB with the hydrophobic coating. The solid yellow line around the recirculation bubble denotes zero velocity (*U* = 0). URB is the upper recirculation bubble, LRB is the lower recirculation bubble, SSB is the slant separation bubble, *RL* is the recirculation length, and *RH* is the recirculation height

The mean flow field over the SAB and EAB with and without the hydrophobic coatings is reported here at the symmetry plane. Figure 4a and b are the contours without the hydrophobic coatings and Figs. 4c and d are the contours with the hydrophobic coatings.

The flow characteristics of a simplified Ahmed body (SAB) without and with a hydrophobic coating are shown in Figs. 4a and b. Table 1 documents the important flow parameters for the models. For the SAB without the hydrophobic coating, the flow separates at the upper edge of the slant surface, creating an SSB that reattaches at 62% of the slant length. Near the lower edge of the slant surface, the flow separates again, forming upper recirculation bubbles (URB) and lower recirculation bubbles (LRB), with the LRB being smaller due to slower underbody flow. The wake recirculation length (*RL*) and height (*RH*) are 85% and 50% of the slant length, respectively. With the hydrophobic coating, the SSB becomes longer and higher, extending to 80% of the slant length. The flow separation patterns at the upper edge of the slant surface and reattachment at the rear end remain similar to the uncoated SAB. However, the recirculation length decreases by 7.6%, indicating a reduced wake recirculation length due to the hydrophobic coating. Previous studies confirm these findings, highlighting the SSB's fundamental nature and the relationship between wake dimensions and drag force [25–27]. Research on hydrofoils and circular cylinders with hydrophobic coatings aligns with these observations, showing that the coating delays flow separation and reduces wake recirculation length [9]. Consequently, the hydrophobic coating alters the flow characteristics, impacting the SSB and reducing the wake recirculation, which can affect the drag force on the SAB.

**Table 1:** Comparison of the slant separation bubble length (*SBL*), wake recirculation length (*RL*), and Reynolds number (*Re*). All the values are in terms of a percentage of the slant length. All the lengths are non-dimensionalized by model height (*h*)

	**SAB**	**SAB** **with coating**	**EAB**	**EAB** **with coating**	**Literature reference**					
*Re* (10^5^)	$0.43$				(1)^a^ $0.08$	(2)^a^ $7.68$	(3)^a^ $7.68$	(4)^a^ $0.62$	(5)^a^ $0.53$	(6)^a^ $11.10$
*SBL*	62	80	-	-	-	77	75	-	-	61
*RL*	85	82	76	80	78	67	-	80	84	84
*RH*	50	42	93	93	-	-	-	-	-	-

(1)^a ^Numerical study by Minguez et al. [28]**,** (2)^a^ Numerical study by Guilmineau [29], (3)^a^ Experimental study by Zhang et al. [30], (4)^a^ Experimental study by Wang et al. [31], (5)^a^ Experimental study by Sellappan et al. [32], (6)^a^ Experimental study by Liu et al. [33]

The velocity contours of the EAB with and without the coating are shown in Figs. 4c and d. Detailed information about the EAB flow features can be found in Siddiqui and Agelin-Chaab [20]. Hence, only relevant observations are discussed here to compare the EAB with and without the coating. Initially, a fully separated flow is found at the slant surface, which eliminates the existence of the SSB. The wake shows only the URB, with the LRB absent. The wake recirculation length is 76% of the slant length (as reported in Table 1). Additionally, there is a significant increase in the height of the wake recirculation, corresponding to an increase of 93% of the slant length in the EAB without the coating. When the coating is applied to the EAB, the flow separates at the upper edge of the slant surface without forming the SSB, similar to the EAB without the coating. The URB is present in the wake as in the EAB without the coating, indicating no effect of the hydrophobic coating on the onset of the separated shear layer from the upper edge of the slant surface. However, a weak LRB is found away from the vertical base at *X* = 1.8, *Z* = 0.25, suggesting that the hydrophobic coating affected the underbody flow, forming a weaker LRB. The wake recirculation length at *Z* = 0.38 remains around 0.60, similar to the EAB without the coating, with no changes in wake recirculation height. These results indicate that the hydrophobic coating in the EAB does not affect the overall mean flow structure. This observation on the 3D bluff body contrasts with findings on two-dimensional (2D) bluff bodies. The hydrophobic coating affected the separated flow in a 2D backward-facing step and increased the wake recirculation length, leading to drag reduction [3].

### Reynolds stresses

**Fig. 5:**
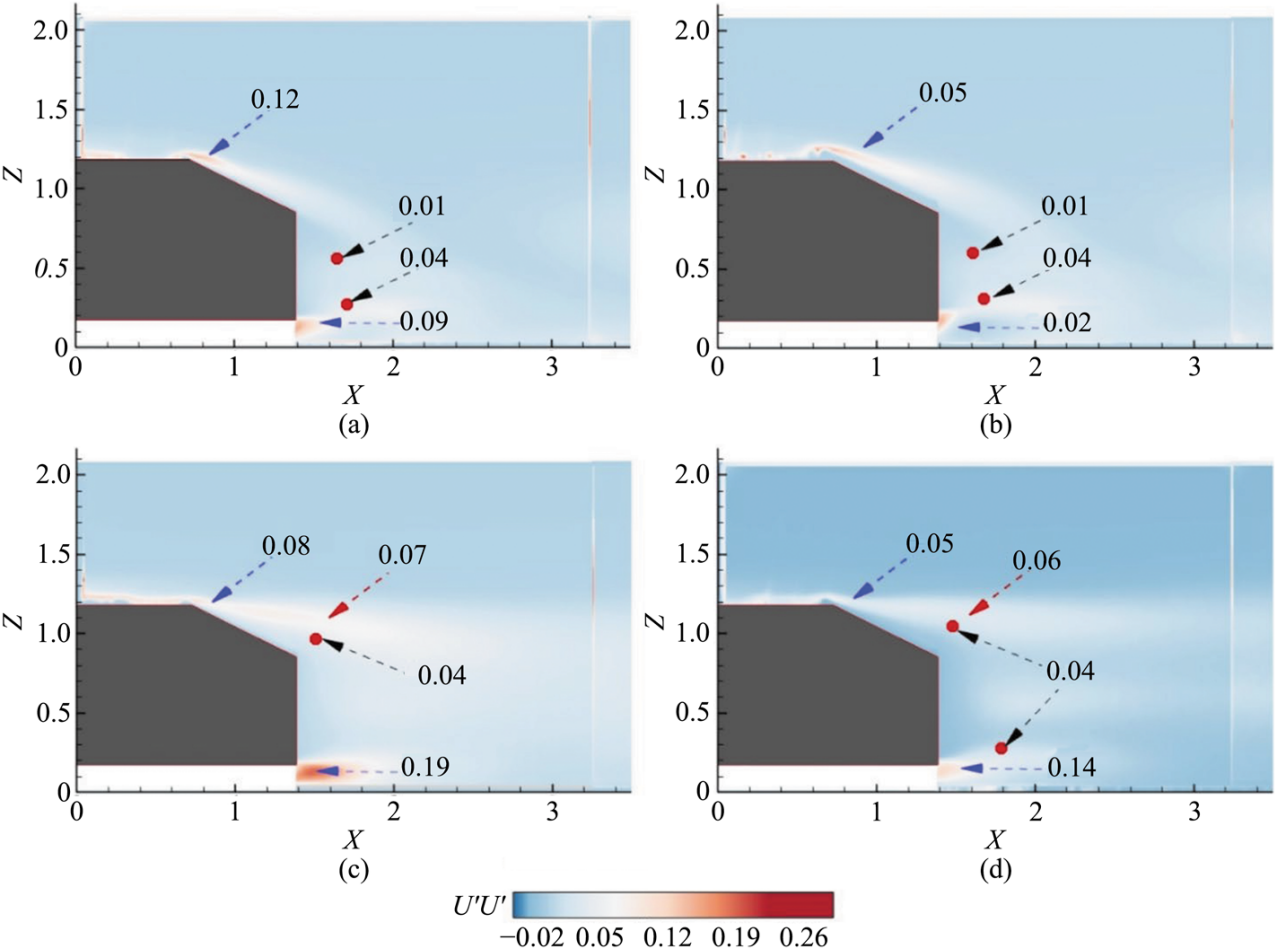
Contours of streamwise Reynolds normal stresses (*U'U'*) at the symmetry plane *Y* = 0. **a** SAB without the hydrophobic coating, **b** SAB with the hydrophobic coating, **c** EAB without the hydrophobic coating, and **d** EAB with the hydrophobic coating. The small solid red circle indicates the center of URB (top) and LRB (bottom)

**Fig. 6:**
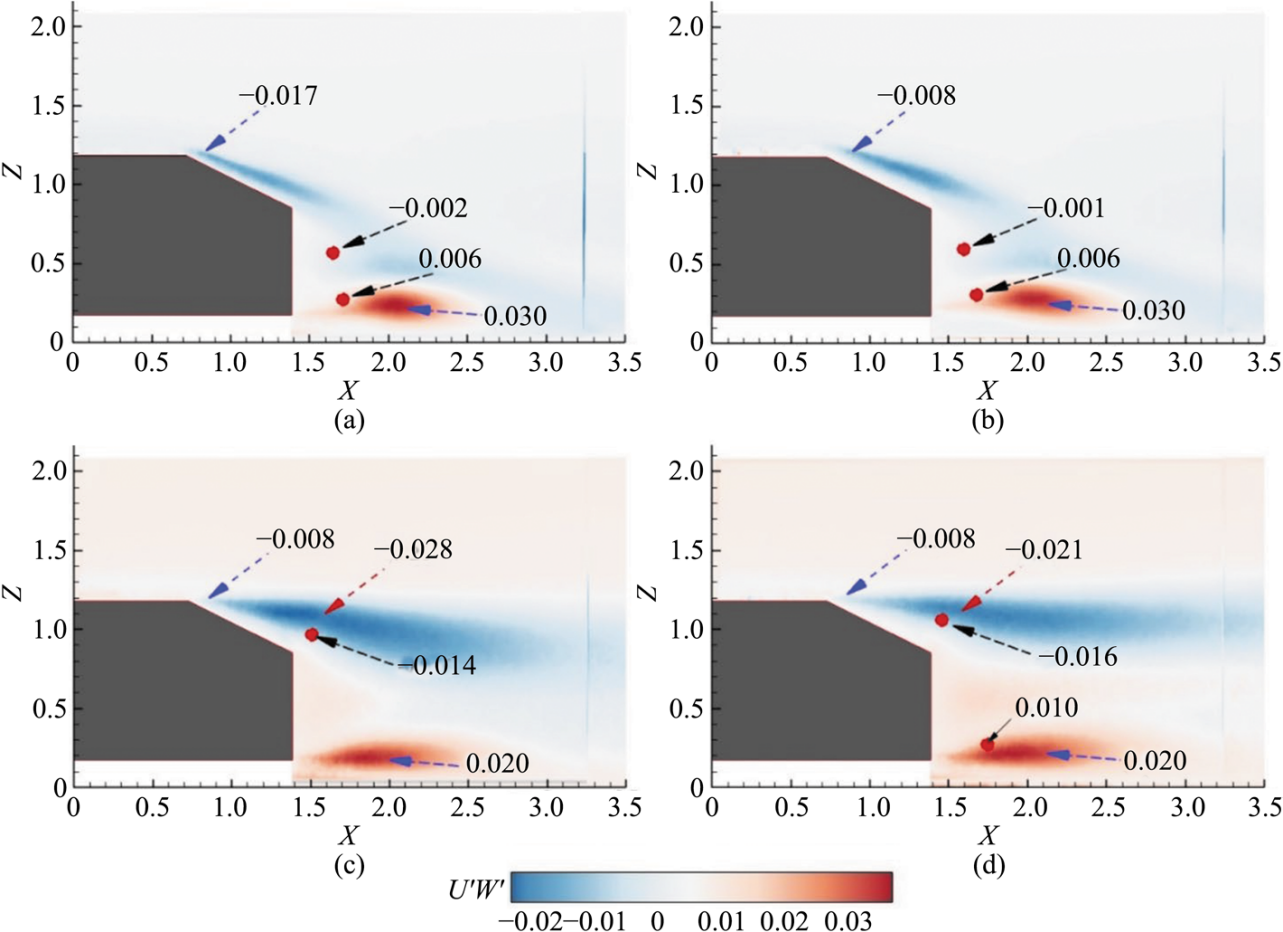
Contours of Reynolds shear stresses (*U'W'*) at the symmetry plane *Y* = 0. **a** SAB without the hydrophobic coating, **b** SAB with the hydrophobic coating, **c** EAB without the hydrophobic coating, and **d** EAB with the hydrophobic coating. The small solid red circle indicates the center of URB and LRB

The effects of the hydrophobic coating on the SAB and EAB are analyzed using the contours of the Reynolds stresses at the symmetry plane. The normal stresses are shown in Fig. 5, and the shear stresses are in Fig. 6.

**Fig. 7:**
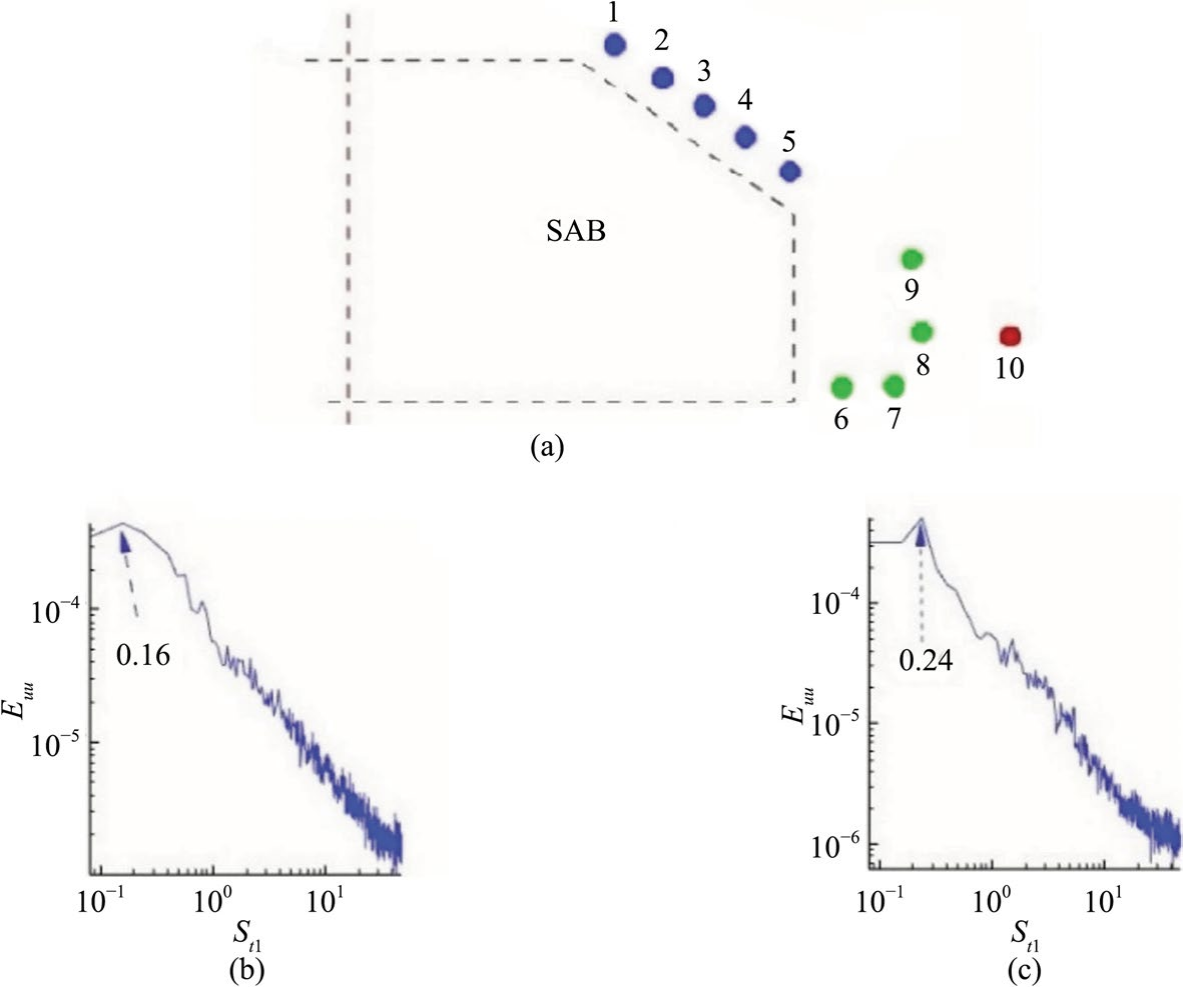
**a** Schematic of the point locations for the Strouhal number extraction for models with and without the hydrophobic coatings, **b** sample of the Strouhal number over the slant surface for SAB without coating, and **c** sample of the Strouhal number over the slant surface for SAB with coating. The locations of the points are indicated in Table 2

The comparison of Reynolds stresses between the SAB with and without the hydrophobic coating reveals several differences, as shown in Figs. 5a and b, 6a and 7b. For the non-coated SAB, two peaks are observed in the *U'U'* contours: one at the top edge of the slant surface (*U'U'* = 0.12) due to flow separation and another at the underbody separated layer (*U'U'* = 0.09). In contrast, with the hydrophobic coating, these *U'U'* values were reduced to 0.05 and 0.02, respectively, although the values in the URB and LRB centers remained unchanged. Regarding the *U'W'* shear stress, the non-coated SAB shows two opposing peaks: a negative peak (*U'W'* = –0.017) along the upper separated shear layer and a positive peak (*U'W'* = 0.03) along the lower underbody shear layer. For the coated SAB, the shear stress over the slant surface decreases to –0.008, while there is no change in the underbody-separated shear layer. Additionally, the URB and LRB centers maintain their negative (*U'W'* = –0.002) and positive (*U'W'* = 0.006) values, respectively, regardless of the coating.

These results suggest that the hydrophobic coating primarily affects the flow structure over the slant surface, reducing shear stress and peak values. However, it does not significantly alter the overall flow structure in terms of URB and LRB characteristics. This trend of reduced normal and shear stresses on superhydrophobic surfaces, contributing to drag reduction, is supported by previous studies [10, 34–37].

Furthermore, the comparison of Reynolds stresses between the EAB with and without the hydrophobic coating reveals notable differences in flow characteristics, as shown in Figs. 5c and d, 6c and 7. Without the coating, the EAB exhibits fully separated flow with *U'U'* peaks = 0.08 in the upper separated shear layer and *U'U'* = 0.19 in the underbody separated shear layer. The *U'W'* shear stresses over the slant surface are lower than *U'W'* = –0.008 compared to the SAB but increase to *U'W'* = –0.028 at later stages of the upper separated shear layer. The URB center shows increased shear stress (*U'W'* = –0.014), indicating a connection with the upper shear layer associated with downwash, although no LRB is present in the EAB. With the hydrophobic coating (Figs. 5d and 6d), the EAB shows comparable *U'U'* values in the upper separated layer and URB, with a slight reduction observed only in the underbody shear layer compared to the uncoated EAB. Similarly, shear stress values in the upper separated shear layer and URB are minimally influenced by the coating (Fig. 6d). The presence of LRB with the coating does not significantly affect shear stress values, suggesting that the hydrophobic coating primarily influences underbody flow while having minimal impact on the fully separated flow characteristics.

Overall, the hydrophobic coating affects the EAB predominantly in the underbody region. Also, slight reductions are observed in the underbody *U'U'* values and minimal changes in the shear stresses compared to the uncoated EAB. This contrasts with the more pronounced changes observed in the SAB, highlighting different effects of the coating on 3D bluff bodies based on the nature of the flow separation.

### Frequency spectra

This section discusses the unsteady flow characteristics of the SAB and EAB with and without the hydrophobic coatings. Strouhal numbers, derived from streamwise velocity using the fast Fourier transformation, are documented in Table 2. Figure 7 illustrates the extraction points for the Strouhal numbers and provides sample frequency spectra.

**Table 2:** Strouhal numbers for the bluff body models with and without the hydrophobic coating

*Re* × 10^5^									
4.3						7.7 (1)^a^	4.6–9.2 (2)^a^	8.9 (3)^a^	0.5–7.0 (4)^a^
Point No #	Locations (*X*, *Z*)	Strouhal #s							
		SAB with coating	EAB with coating	SAB without coating	EAB without coating	SAB without coating (Literature)			
#1	0.84, 1.15	0.24	0.48	0.16	0.88	0.25	0.11	0.35	0.20
#2	0.96, 1.09	0.24	0.48	0.16	0.65	0.21	0.11	-	
#3	1.07, 1.04	0.24	0.55	0.16	0.95	0.21	0.11	-	-
#4	1.17, 0.99	0.24	0.40	0.16	0.55	0.15	-	0.27	-
#5	1.31, 0.95	1.8	0.91	0.30	0.70		-	-	-
#6	1.46, 0.12	0.48	0.62	0.55	0.50	0.45	0.53	-	0.44
#7	1.66, 0.13	0.23	0.85	0.31	0.46	-	-	-	
#8	1.72, 0.28	0.16	0.25	0.55	0.50	-	-	-	
#9	1.65, 0.57	0.66	0.80	0.61	0.23	-	-	-	
#10	2.05, 0.35	1.19	0.48	0.56	0.48	0.45	-	0.42	

(1)^a^ by Delassaux et al. [38], (2)^a^ by Thacker et al. [39], (3)^a^ by Minguez et al. [40], and (4)^a^ by Zhang et al. [30]

The Strouhal numbers (*S*_*t*_) between the SAB with and without the hydrophobic coating are shown in Table 2. Without the coating, the SAB exhibits a dominant *S*_*t*_ = 0.16 over the slant surface, consistent with previous experimental findings by Zhang et al. [30]. A higher peak at *S*_*t*_ = 0.3 near the rear edge of the slant surface is also observed, aligning with reported values in the literature. In the wake, *S*_*t*_ values vary, with peaks around *S*_*t*_ = 0.55 near the model's base and *S*_*t*_ = 0.61 at the centers of the URB and LRB, comparable to studies in [30, 38]. They suggest quasiperiodic ordered structures alternately emitted from the URB and LRB in the wake at this *S*_*t*_. With the hydrophobic coating, the SAB shows altered *S*_*t*_ values primarily over the slant surface. A consistent *S*_*t*_ = 0.24 is observed, about 33% higher than without the coating, except at the rear edge, where different values occur. In the wake, *S*_*t*_ values remain within a similar range as the uncoated SAB, indicating minimal impact from the coating on wake dynamics. This increase in *S*_*t*_ over the slant surface with the coating is attributed to modifications in the SSB, delaying separation at the rear edge. However, after separation, the coating does not significantly alter the overall wake behavior. The literature on bluff bodies, such as elongated bodies and spheres with hydrophobic coatings, similarly reports an increase in Strouhal numbers [3, 41]. This highlights a common trend of enhanced wake dynamics due to the coating's effects on flow separation and shedding patterns.

Comparing the Strouhal numbers between the EAB with and without the hydrophobic coatings reveals distinct effects on flow dynamics documented in Table 2. Without the coating, the EAB exhibits higher *S*_*t*_ values over the slant surface, notably *S*_*t*1_ = 0.88 near the point of flow separation and *S*_*t*5_ = 0.70 at the rear end, indicating variability without a dominant frequency. In the lower shear layer (points #6 and #7), *S*_*t*_ values are similar to those of the uncoated SAB. Interestingly, despite the absence of an LRB, higher *S*_*t*8_ = 0.50 is observed at its location, while *S*_*t*9_ = 0.23 is comparatively lower. *S*_*t*10_ values remain comparable to the uncoated SAB, suggesting a shift in URB influencing higher slant surface *S*_*t*_ values while wake frequencies are less affected.

With the hydrophobic coating, the EAB shows an average *S*_*t *_≈ 0.47 over the slant surface (points #1 to #4). This is a slight reduction compared to the uncoated scenario, but with an increased *S*_*t* _≈ 0.91 at the rear end, similar to the uncoated EAB. In the wake, varied frequencies include *S*_*t*6 _≈ 0.62 and *S*_*t*7 _≈ 0.85 near the underbody separated shear layer, *S*_*t*8 _≈ 0.25 at point #8, and *S*_*t*9 _≈ 0.80 at point #9, with *S*_*t*__10 _≈ 0.48 remaining consistent. This indicates a nuanced effect of the coating, reducing slant surface *S*_*t*_ values while introducing higher and lower frequencies in the wake compared to the uncoated EAB. Despite the minimal impact on the time-averaged flow structures, the hydrophobic coating influenced the unsteady flow characteristics of the EAB, aligning with its effect on flow separation dynamics.

### Proper orthogonal decomposition

The proper orthogonal decomposition (POD) is a model decomposition method in which flow fields are classified into orthogonal spatial modes. The current paper used the same method of POD employed previously by the authors in Siddiqui and Agelin-Chaab [20], and therefore, a detailed description is not repeated here. The POD is extracted based on the instantaneous streamwise velocity using the snapshot method. This study uses the POD to extract the dominant frequency in the wake of the models. It is calculated based on the method used by Thacker et al. [42]. They reported that a wavelength could be calculated between alternate positive–negative regions of velocity and using the convection velocity as ${U}_{o}=0.5\times {U}_{\infty }$, the frequency of these alternate velocities can be found with the $f={U}_{o}/\lambda$ relation.

**Fig. 8:**
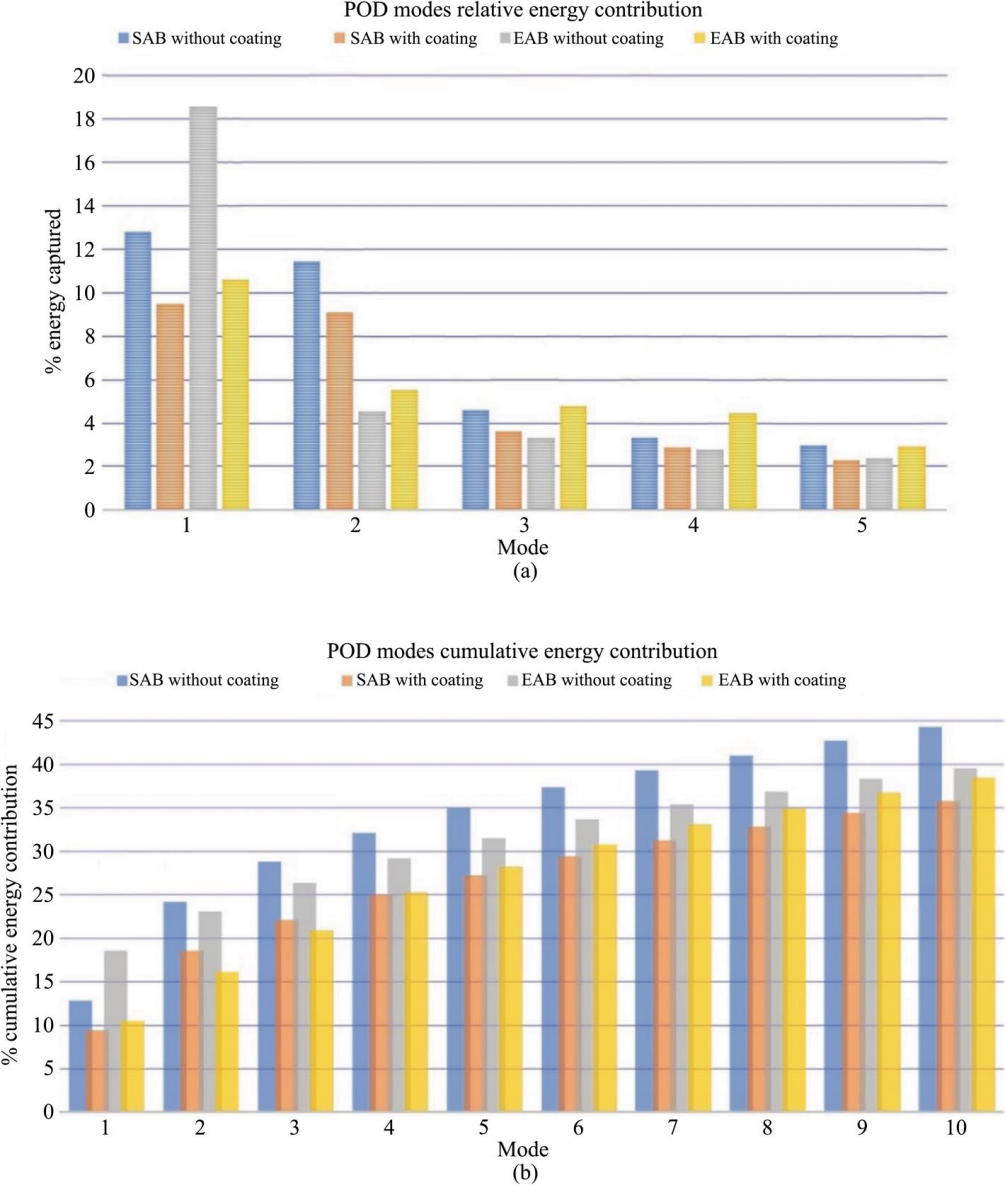
**a** Relative and **b** cumulative energy extracted from the POD eigenvalues in the wake region

At first, Figs. 8a and b show the relative energy in the first five modes and the accumulative energy within the initial ten modes. The POD was extracted within identical regions of the same size for all cases within the following limits: {*X* = 1.15–3.3} and {*Z* = 0.025–2.05}. According to Fig. 8a, the relative energy captured in the 1st and 2nd POD modes in the SAB without the hydrophobic coating is 12.8% and 11.4%, respectively, and this drops to 4.5% in the 3rd mode. On the other hand, for the SAB with the hydrophobic coating, the 1st and 2nd modes have 9.4% and 9% relative energy, respectively, but drops to 3.5% in the 3rd mode. The lower values suggest a redistribution of the energy in the lower modes due to the hydrophobic coating. In addition, the EAB without the hydrophobic coating shows a higher relative energy of 18.5% in the 1st POD mode but only 4.5% in the 2nd mode. The corresponding values for the EAB with the hydrophobic coating are 10.5% and 5.5% for the 1st and 2nd modes, respectively. It also suggests a significant redistribution of the energy due to the hydrophobic coating. In general, it can be observed from Fig. 8a that fractional energy from the 1st POD mode to the 5th decays more steeply for both the SAB and EAB without the hydrophobic coatings than with the coatings. Furthermore, the cumulative energy in Fig. 8b shows an increasing trend, as expected. It is observed that the first 10 modes contribute to about 44% of energy in the SAB without the hydrophobic coating, whereas the SAB with the hydrophobic coating contributes 36%. On the other hand, in the EAB with and without the hydrophobic coating, the cumulative energy is closer to 38% and 40%, respectively. The results support the authors’ hypothesis of modal energy redistribution due to the hydrophobic coatings.

**Fig. 9:**
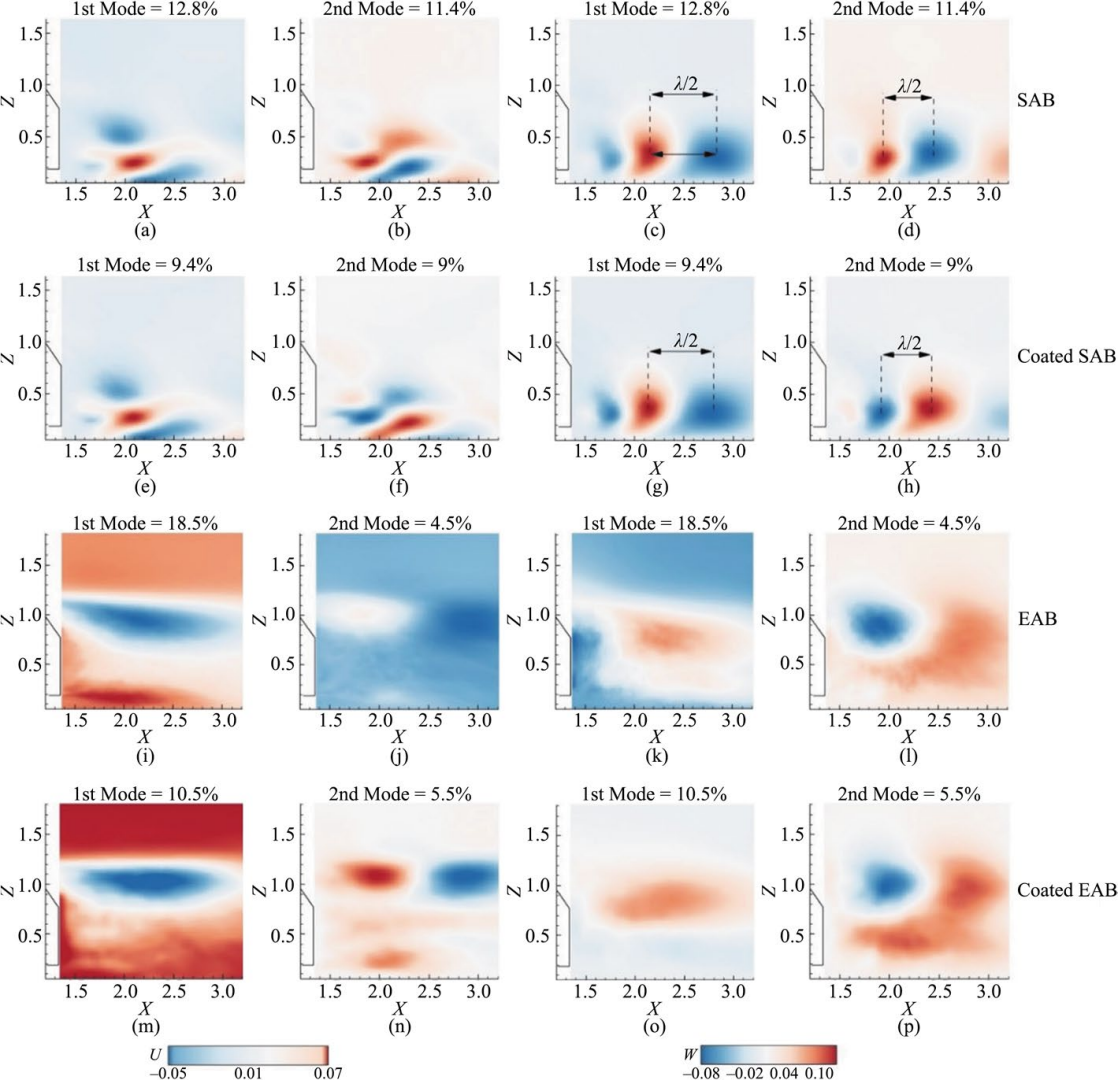
Velocity contours of the POD modes. SAB without the hydrophobic coating: **a–b** Streamwise velocity and **c–d** wall-normal velocity; SAB with the hydrophobic coating: **e–f** Streamwise velocity and **g–h** wall-normal velocity; EAB without the hydrophobic coating: **i–j** Streamwise velocity and **k–l** wall-normal velocity; EAB with the hydrophobic coating: **m–n** Streamwise velocity and **o–p** wall-normal velocity

The streamwise and wall-normal velocity contours of the 1st and 2nd POD modes for all the models are shown in Fig. 9. The streamwise and wall-normal velocity contours of SAB without the hydrophobic coating are shown in Figs. 9a–b and c–d, whereas those with the hydrophobic coating are displayed in Figs. 9e–f and g–h, respectively. In the present study, the 1st and 2nd modes were used to obtain the frequencies, from which the Strouhal numbers were estimated based on *S*_*t*_ = *fh*/*U* (where *h* is model height and *U* freestream velocity). For example, frequencies of $f=$ 0.33 and 0.48 Hz, and hence about *S*_*t*_ = 0.36 and 0.48 were obtained in the SAB without the hydrophobic coating. These *S*_*t*_ values are consistent with those reported in Table 2. Also, the *S*_*t*_ values correspond to *S*_*t*_ = 0.33, as reported by Uruba and Dynamics [43] for the 1st and 2nd modes of the SAB without the hydrophobic coating. Furthermore, similar Strouhal numbers are obtained for the SAB with the hydrophobic coating corresponding to 1st and 2nd modes approximately *S*_*t*_ = 0.36 and 0.48 based on Figs. 9e–f and g–h. It suggests that the hydrophobic coating has not significantly affected the coherent structures. This may be due to the fact that the effect of the hydrophobic coating is most significant on the slant surface flow. On the other hand, no dominant Strouhal number is observed in the wake (Figs. 9i–p) for the EAB with and without the hydrophobic coating.

### Dynamic mode decomposition

The dynamic mode decomposition (DMD) is a dimensionality-reduction technique which provides a spatiotemporal decomposition of data into dynamic modes derived from snapshots or measurements of a system in time. As a result, extracting the inherent frequencies associated with the DMD modes is possible. However, the selection of a mode to represent the dynamics involved is a cumbersome issue and has been debated in the literature, leading to several DMD variations [44]. Nonetheless, for the current study, the DMD in the wake of all four models is used to capture the dominant frequencies in the wake. The DMD modes can be obtained by using a time series of measurement snapshot vectors, ${x}_{n}$ at each time step $n$ are arranged in a matrix ${X}_{1}^{N}\leftarrow \{{x}_{1},{x}_{2},\dots \dots {x}_{N}\}$. The data matrix ${X}_{1}^{N}$ is decomposed into two sets: ${X}_{1}^{N-1}\leftarrow \{{x}_{1},{x}_{2},\dots \dots {x}_{N-1}\}$ and ${X}_{2}^{N}\leftarrow \{{x}_{2},{x}_{2},\dots \dots {x}_{N}\}$. The first set is orthogonalized using the singular value decomposition (SVD): $\left[U,\Sigma ,W\right]=svd({X}_{2}^{N-1})$, where *U* includes the proper orthogonal modes of the first set of data ${X}_{1}^{N-1}$. It is thus possible to express the last data vector ${x}_{N}$ as a linear combination of the previous elements in the form [45]

10 $$S={U}^{H}{V}_{2}^{N}W{\Sigma }^{-1}.$$

Model structures are represented by the eigenvectors of *S*, whereas frequencies and growth/decay rates are represented by the eigenvalues. In order to quantify how many modes representation there is in the original dataset, the optimum amplitudes can be computed by QR-decomposition of the original data matrix *V*, the singular values $\Sigma$, and the modes (Eigenvectors of *S*). The discrete-time eigenvalue is used to study the stability characteristics of the DMD modes. The eigenvalues occur as complex conjugate pairs and lie on a unit circle in the complex domain representing the modes with zero growth rates. However, the eigenvalues lying inside the unit circle represent the decaying of DMD modes, and outside existence shows growth with time [46]. The DMD provides a Strouhal number based on the equation below:

11 $$(St = f_i \ast h/U_{\infty}).$$

**Fig. 10:**
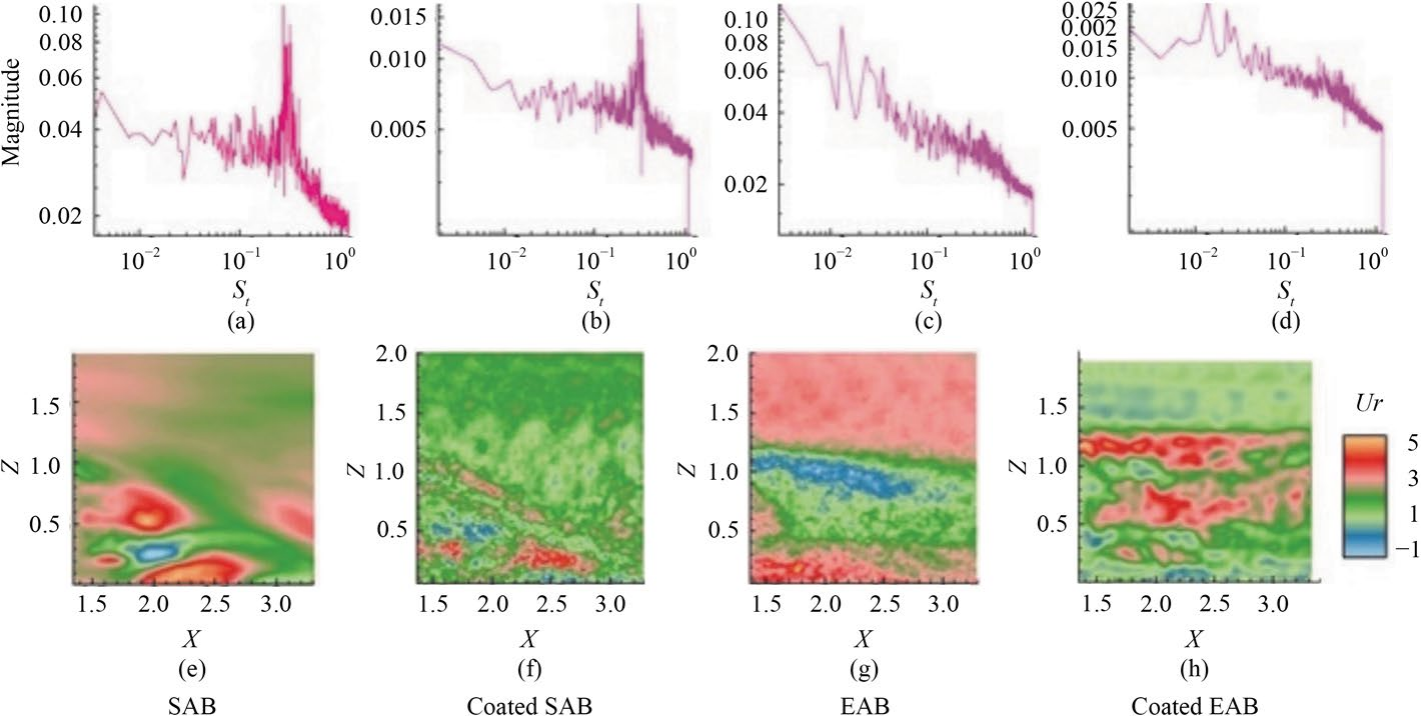
DMD analysis of the models. The first row indicates the dominant Strouhal number found in the wake of the model, and the second row shows the corresponding contours of the real part of the streamwise velocity. Here **a** and **e** SAB without the hydrophobic coating, **b** and **f** SAB with the hydrophobic coating, **c** and **g** EAB without the hydrophobic coating, and **d** and **h** EAB with the hydrophobic coating

In Eq. (11),* f*_*i*_ = 2πIm(log(*λi*))/Δ*t* with *λi* as the complex eigenvalues, *h* being the model height, and *U*_∞_ is the velocity. The Strouhal number of all the models with the associated DMD modes is shown in Fig. 10. For this study, we selected DMD modes based on their eigenvalues and modal amplitudes, focusing on modes with significant contributions to the overall dynamics while ensuring physical relevance. This approach is consistent with the literature and ensures a robust representation of the wake dynamics for all four models.

Based on Fig. 10, the dominant Strouhal number in the SAB without the hydrophobic coating is *S*_*t*_ = 0.27, and in the SAB with the hydrophobic coating, *S*_*t*_ = 0.30. These Strouhal numbers are close to the values found in the POD analysis. The corresponding DMD modes show alternate positive and negative velocity contours in Fig. 10a for the SAB without the coating. However, the SAB with the hydrophobic coating does not show the presence of such an alternate vortex structure in Fig. 10f. The dominant Strouhal number for the EAB, with and without the hydrophobic coating, is close to *S*_*t*_ = 0.013. The corresponding DMD modes show energetic velocity contours in Figs. 10g–h. According to the literature, a lower DMD Strouhal number suggests a more energetic mode and a higher one indicates a less energetic [47]. These low Strouhal numbers suggest the existence of a large-scale energetic structure having higher energy, which can be seen in Figs. 10g–h. The POD analysis supports this explanation since it captured the higher energy of 18.5% in the 1st POD mode of EAB.

The dominant Strouhal number for the SAB without the hydrophobic coating, *S*_*t*_ = 0.27, and for the SAB with the hydrophobic coating, *S*_*t*_ = 0.30, aligns with some of the pointwise Strouhal numbers obtained through frequency spectra analysis, as shown in Fig. 7 and Table 2. However, when averaging the wake Strouhal numbers from frequency spectra analysis, the value is approximately *S*_*t*_ = 0.52 for all models, regardless of the coating. In contrast, DMD revealed an averaged dominant Strouhal number of around *S*_*t*_ = 0.28 for the SAB models and *S*_*t*_ = 0.013 for the EAB models. The divergence between these results stems from the well-known distinct methodologies and focuses of these techniques. As pointed out before, DMD identifies dominant modes based on eigenvalues, emphasizing global flow features, particularly large-scale, energetic structures associated with slower oscillations. Frequency spectra analysis, on the other hand, captures a broader frequency range, including high-frequency fluctuations commonly seen in turbulent wakes. This difference is further amplified by spatial averaging in frequency spectra analysis, which highlights localized high-frequency activity, contrasting with DMD’s focus on global coherence. Additionally, time-windowing effects contribute to the discrepancy. DMD assumes linear evolution of modes over the selected time window, which tends to smooth out transient, high-frequency phenomena that frequency spectra analysis captures effectively. Finally, the choice of specific measurement locations in frequency spectra analysis also amplify high-frequency contributions, further increasing the observed differences between the two approaches. Due to such differences the current study used both methods to compare and contrast the dominant frequencies and add valuable information to the existing literature [48].

## Conclusions

This study explored how hydrophobic coatings affect the intricate flow patterns of two 3D bluff bodies, specifically focusing on flow separation characteristics. Two variants of the Ahmed body were studied: the standard Ahmed body (SAB), featuring flow separation and reattachment at its slant surface, and the elliptical Ahmed body (EAB), which exhibits fully separated flow. Experiments involved both coated and uncoated versions of the SAB and EAB, employing time-resolved and standard particle image velocimetry to measure velocities at a Reynolds number of 4.31 × 10^4^ based on the model height. The following observations can be summarized from the results obtained for the SAB and EAB without the hydrophobic coating:

- The mean flow features of the SAB show a three-dimensional flow structure at the rear end, having a slant separation bubble, longitudinal C-vortices, and a wake recirculation region.
- On the other hand, the EAB provides a fully separated flow at the rear end, which eliminates the lower recirculation bubble.
- The single predominant Strouhal number of *S*_*t*_ = 0.16 exists over the slant surface of the SAB, which is associated with the flapping of the slant separation bubble.
- In contrast, due to the fully separated flow in the EAB, an increased Strouhal number of *S*_*t*_ = 0.70–0.88 was observed at the slant surface that can be ascribed to the detached shear layers.
- In the wake of the SAB, values of *S*_*t*_ = 0.30–0.61 were estimated using the frequency spectra analysis. These can be associated with the quasiperiodic phenomena related to the alternate emanation of fluid from the URB and LRB.
- Values of *S*_*t*_ = 0.23–0.5 were observed in the wake of the EAB, but it should be noted that, in this case, the wake contained only the URB and not LRB.
- The proper orthogonal decomposition (POD) revealed a dominant Strouhal number associated with the 1st and 2nd POD modes of *S*_*t*_ = 0.36 and *S*_*t*_ = 0.48, respectively, in the SAB. On the other hand, no such Strouhal numbers are found in the wake of the EAB.
- The dynamic mode decomposition (DMD) also provides a dominant *S*_*t*_ = 0.27 for the SAB, whereas the EAB shows a low *S*_*t*_ = 0.013. Such a low Strouhal number suggests that the frequency of the wake flow structure is reduced, and a higher energy spatial mode exists with lower *S*_*t*_ values.

In contrast, the following observations are made for the SAB and EAB with the hydrophobic coating:

- The hydrophobic coating affects the SAB more significantly than the EAB. This is because the existence of the slant separation bubble is affected more by the hydrophobic coating than the fully separated flow in the EAB.
- The slant separation bubble of the SAB is modified, and the reattachment length is increased by 80% of the slant length, leading to a delay in the secondary separation.
- A reduction in the shear stresses is also observed in the SAB. Furthermore, the Strouhal number increases in the SAB, and a dominant value of *S*_*t*_ = 0.24 is found at the slant surface compared to the SAB without the hydrophobic coating.
- In the EAB with the hydrophobic coating, the Strouhal number over the slant surface is in the range of *S*_*t*_ = 0.40–0.55.
- In the wake of the EAB with the hydrophobic coating, the DMD analysis showed a dominant Strouhal number of *S*_*t*_ = 0.013, which suggests the existence of energetic modes. Whereas, in the POD analysis, such low Strouhal numbers were not observed.
- The POD shows the Strouhal number for the 1st and 2nd modes as *S*_*t*_ = 0.36 and *S*_*t*_ = 0.48, respectively, in the wake of the SAB.
- The POD analysis also revealed that the hydrophobic coatings redistributed the energy in the low modes so that the fractional energy has a steeper decay from the 1st POD mode to the 5th for both the SAB and EAB.
- The DMD analysis revealed a dominant Strouhal number of *S*_*t*_ = 0.3. Both the POD and DMD analyses provide similar values of the dominant Strouhal number in the wake of the SAB.

Based on the findings above, it is evident that the impact of the hydrophobic coatings varied significantly between the 3D Ahmed body models due to their distinct rear-end flow separation characteristics. The study highlights that hydrophobic coating exerted a more pronounced influence on the bluff bodies that are characterized by separation and reattachment on the rear slant surface, compared to those with fully separated flow at the rear end. The observed changes in flow patterns due to the hydrophobic coatings suggest potential for drag reduction, specifically in the SAB configuration. While this study provides valuable insights into the effects of hydrophobic coatings on these bluff bodies, further research is needed to optimize surface roughness before definitive conclusions can be drawn. Additionally, future investigations should focus on the entire rear-end flow structure beyond the symmetry plane and quantify drag effects more comprehensively. Furthermore, understanding how Reynolds number impacts the effectiveness of hydrophobic coatings in altering flow structures is crucial for advancing this research area.

## Data Availability

The supporting data is available from the authors on reasonable request.

## Nomenclature

DF-PIV: Double frame particle image velocimetry
DMD: Dynamic mode decomposition
EAB: Elliptical Ahmed body
*E*_*uu*_: Energy spectrum
FOV: Field of view
FFT: Fast Fourier transformation
*g*: Acceleration due to gravity
*h*: Test model height
*H*: Water depth
*Hr*: Recirculation height
HPS: Hydrophobic surfaces
IA: Interrogation area
IDDES: Improved delayed detached eddy simulation
*Lr*: Recirculation length
LRB: Lower recirculation region
LSV: Large-scale separated vortex
POD: Proper orthogonal decomposition
PIV: Particle image velocimetry
QAS: Quasi axisymmetric structure
*R*_*uu*_: Streamwise auto-correlation
SSB: Slant separation bubble
SAB: Standard Ahmed body
SVD: Singular value decomposition
S-PIV: Standard particle image velocimetry
*S*_*t*_: Strouhal number
*S*_*k*_: Stokes number
*SL*: Slant length
SHPS: Superhydrophobic surfaces
TDS: Three-dimensional structure
TKE: Turbulent kinetic energy
TR-PIV: Time-resolved particle image velocimetry
*t*_*p*_: Particle response time
*t*_*f*_: Characteristic temporal scale
URB: Upper recirculation region
*U*: Normalized time-averaged streamwise velocity
*U*_∞_: Freestream velocity
*U’U’*: Normalized normal stress
*U’W’*: Normalized shear stress
*V*: Normalized time-averaged spanwise velocity
*W*_*r*_: Recirculation width
*W*: Test Model width
*X*: Normalized streamwise coordinates
*Y*: Normalized spanwise coordinates
*Z*: Normalized wall-normal coordinates
*W*: Normalized time-averaged wall-normal velocity
*α*: Slant angle
*ν*: Viscosity
*ρ*: Density
*ρ*_*f*_: Density of the seeding particle
*ρ*_*p*_: Density of the fluid
